# Recent Advances in our Understanding of Tocopherol Biosynthesis in Plants: An Overview of Key Genes, Functions, and Breeding of Vitamin E Improved Crops

**DOI:** 10.3390/antiox6040099

**Published:** 2017-12-01

**Authors:** Steffi Fritsche, Xingxing Wang, Christian Jung

**Affiliations:** 1 Plant Breeding Institute, Christian-Albrechts-University of Kiel, 24118 Kiel, Germany; wangxingxing@caas.cn (X.W.); c.jung@plantbreeding.uni-kiel.de (C.J.); 2 Institute of Cotton Research of Chinese Academy of Agricultural Sciences, Anyang 455000, China

**Keywords:** vitamin E, tocopherol, antioxidant, biofortification, crop breeding

## Abstract

Tocopherols, together with tocotrienols and plastochromanols belong to a group of lipophilic compounds also called tocochromanols or vitamin E. Considered to be one of the most powerful antioxidants, tocochromanols are solely synthesized by photosynthetic organisms including plants, algae, and cyanobacteria and, therefore, are an essential component in the human diet. Tocochromanols potent antioxidative properties are due to their ability to interact with polyunsaturated acyl groups and scavenge lipid peroxyl radicals and quench reactive oxygen species (ROS), thus protecting fatty acids from lipid peroxidation. In the plant model species *Arabidopsis thaliana*, the required genes for tocopherol biosynthesis and functional roles of tocopherols were elucidated in mutant and transgenic plants. Recent research efforts have led to new outcomes for the vitamin E biosynthetic and related pathways, and new possible alternatives for the biofortification of important crops have been suggested. Here, we review 30 years of research on tocopherols in model and crop species, with emphasis on the improvement of vitamin E content using transgenic approaches and classical breeding. We will discuss future prospects to further improve the nutritional value of our food.

## 1. Introduction

Four tocopherols and four tocotrienols form a group of lipid-soluble antioxidants called tocochromanols, commonly known as vitamin E [1,2]. Their basic structure is simple, comprising a polar chromanol ring and a hydrophobic polyprenyl side chain, products of the shikimate and 1-deoxy-d-xylulose 5-phosphate (DOXP) pathways. Tocochromanols with a fully saturated side chain are called tocopherols and those with an unsaturated side chain tocotrienols. The number of methyl groups in the chromanol ring defines the four natural occurring α, β, γ, and δ-tocopherol and tocotrienol subforms [3]. Vitamin E activity involves scavenging peroxyl radicals and quenching reactive oxygen species (ROS). The most active form of vitamin E is α-tocopherol and thus the most needed form of vitamin E in our diet [4]. It is generally found in high concentrations in vegetable oils such as almond, safflower, or canola oil or in other high-fat sources such as nuts, seeds, or grains [5]. Food-grade canola oil, or 00-type rapeseed oil, has a high-quality nutritional composition similar to that of olive oil, and tocopherols are one of the main nutritionally relevant constituents. Despite vitamin E’s importance in the human diet, dietary studies show that the recommended daily allowance is often not met, so, improving its quantity and composition has become a target in crop breeding [6].

In this review, we follow the elucidation of the tocopherol biosynthesis pathway genes over the last 30 years of research, with a particular focus on their function and their involvement in plant processes. We will focus on the biosynthesis bottlenecks detected and the transgenic and non-transgenic breeding efforts that have been undertaken in order to improve the antioxidant and nutritional values of important crops.

## 2. Biosynthesis and Chemical Function

**Figure 1:**
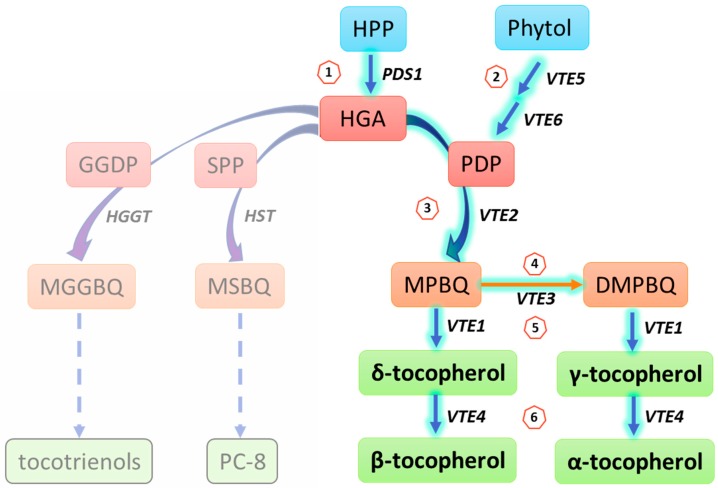
Simplified model of the tocopherol biosynthesis pathway in *A. thaliana* (highlighted branch of the pathway). Compound names are given in the boxes and gene names are indicated in italic. The circled numbers refer to the biosynthesis steps described in detail in Table 1. Adapted from [15]. *HGGT*: homogentisic acid geranylgeranyl transferase; *HST*: homogentisic acid solanesyl transferase; GGDP: geranylgeranyl diphosphate; MGGBQ: 2-methyl-6-geranylgeranyl-1,4-benzoquinol; PC-8: plastochromanol-8; SPP: solanesyl pyrophosphate.

Tocopherol biosynthesis is initiated in the plant’s cytoplasm, but, except for this first step, its biosynthesis takes place in the plastids. Here, the necessary enzymes are localized at the inner envelope or in the plastoglobules [7,8,9]. The biosynthesis starts through the formation of the aromatic head group homogentisic acid (HGA), which is catalyzed by the enzyme *p*-hydroxyphenylpyruvate and is derived from tyrosine degradation [10,11]. The polyprenyl side chain, phytyl diphosphate (phytyl-PP/PDP), is suggested to originate from the DOXP pathway, as well as from the recycling of free phytol derived from the chlorophyll degradation process [12,13]. In *Arabidopsis thaliana (A. thaliana)*, this step is catalyzed sequentially by two phytol kinases [13,14]. The two substrates, HGA and PDP, are fused together in the following step, mediated by the enzyme homogentisate phytyltransferase (HPT), to 2-methyl-6-phytyl-1,4-benzoquinol (MPBQ). MPBQ, in turn, serves as substrate either for tocopherol cyclase (TC) or MPBQ methyltransferase. MPBQ methyltransferase methylates MPBQ to 2,3-dimethyl-6-phytyl-1,4-benzoquinone (DMPBQ), while tocopherol cyclase transforms both MPBQ and DMPBQ to γ- and δ-tocopherol, respectively. Lastly, the enzyme γ-tocopherol methyltransferase (γ-TMT) catalyzes the conversion of γ- to α-tocopherol and of δ- to β-tocopherol (Figure 1, Table 1).

Tocopherols are one of the most valuable antioxidants because of their remarkable chemical mode of action. They interact with polyunsaturated acyl groups and protect polyunsaturated fatty acids from lipid peroxidation by scavenging lipid peroxy radicals and quenching ROS produced e.g., by photosystem II and during membrane lipid peroxidation [34,35]. During this process, tocopherols donate their phenolic hydrogen to lipid-free radicals, thus neutralizing the radical, terminating the autocatalytic lipid peroxidation processes and protecting cell membranes [36,37]. The resulting tocopherol radicals are more stable, are less reactive, and, more importantly, can be reconverted to the corresponding tocopherol by reacting with other antioxidants such as ascorbate or glutathione. This allows each tocopherol to participate in many scavenging reactions before being degraded. Collectively, these properties make tocopherols highly efficient as antioxidants [38]. Furthermore, tocopherols are able to deactivate singlet oxygen (^1^O_2_), which oxidizes, amongst others, membrane lipids, proteins, amino acids, nucleic acids, nucleotides, and carbohydrates [39,40]. The chemical reaction of tocopherols with ^1^O_2_ results in the corresponding tocopherol quinones (and other derivatives), some of which have been shown to be potent antioxidants [41,42,43].

## 3. Tocopherols in Plants and Mammalians

Tocopherols occur in mammalians, photosynthetic bacteria, fungi, algae, and plants, but only photosynthetic organisms are able to synthesize them [2,3]. The content, composition, and presence of tocopherols varies widely in different plant tissues. They can be found in seeds, fruits, roots, and tubers and are usually present in the green parts of higher plants [15,44]. The most abundant form in leaves is α-tocopherol, whereas the dominating tocopherol form in seeds is γ-tocopherol [2,33]. Nevertheless, in some crops, for instance, sunflower, olive, safflower, wild *Euphorbia*, or grape, α-tocopherol is the main tocopherol form in seeds [45,46,47,48,49]. Total tocopherol content and composition strongly changes under conditions of plant oxidative stress including high light, salinity, drought, and low temperatures [3,31,32,50,51]. Both plant growth and development affect the levels of tocopherol content and composition, which changes for example during senescence, chloroplast to chromoplast conversion, fruit ripening, and seed development [51,52,53,54,55].

Since 1922, when the term vitamin E was introduced by Evans and Bishop [56], the role of vitamin E has been extensively studied in mammalian systems and the beneficial effects demonstrated. Their antioxidant and radical scavenging mechanisms have been described in a variety of studies [57,58,59,60]. In these studies, tocopherols and tocotrienols are termed vitamin E as they usually occur as a mixture in food. Because vitamin E cannot be synthesized by humans, it is an essential component of our nutrition. A sufficient uptake of vitamin E can help to prevent neurological disorders and chronic diseases, especially those believed to have an oxidative stress component such as atherosclerosis, cataracts, and cancer [60,61,62,63,64,65,66,67,68,69]. In recent years, studies focused also on the role of other vitamin E forms such as γ-tocopherol, δ-tocopherol, and γ-tocotrienol as well as on the non-antioxidant molecular mechanisms to explain the regulatory effects of vitamin E on signal transduction and gene expression in mammals [70,71,72].

According to the German Society for Nutrition (Deutsche Gesellschaft für Ernährung e.V.), the recommended daily amount of vitamin E for an adult between 25 and 51 years is 14 mg/day. Nuts, seeds, and vegetable oils are among the best sources for the dietary uptake but also green leafy vegetables, fruits, and cereals. Soybean, rapeseed, and corn oil are the best dietary sources for γ-tocopherol, whereas the most biologically active form of vitamin E, α-tocopherol, can be found in wheat germ oil, sunflower, and olive oils [34,73]. Of the naturally occurring α-forms, the stereoisomers RRR-α-tocopherols have the highest biological activity. They can be stored and transported in the body due to specific selection by the hepatic α-tocopherol transfer protein (α-TTP) [34]. Thus, the increase in vitamin E content and the enrichment of α-tocopherol in vegetable oils is of particular interest in crop breeding.

## 4. Studying the Plant Tocopherol Biosynthesis Key Genes 

In leaves, tocopherols are located in chloroplast membranes, plastoglobules, and thylakoid membranes and are thus in close proximity to the photosynthetic apparatus. For this reason, it was believed that tocopherols were the most important antioxidants in plants for preventing damage to the apparatus from photosynthesis-derived ROS. However, recent studies indicate that tocopherols only partially contribute to a considerable variety of plant antioxidants such as carotenoids, ascorbates, glutathiones, and flavonoids, which also contribute to photo-protection [74,75,76]. Nevertheless, tocopherols are crucial for the inhibition of non-enzymatic lipid peroxidation during stress conditions, supporting the germination of seedlings and are needed for the protection of seed storage lipids [15,32,77]. They are involved in a plethora of others functions such as the activation of plant defense responses, intracellular signaling, transcript regulation, and function in membrane stability [32,77,78,79].

Step 1: Homogentisic Acid Synthesis

The first committed step of the tocopherol biosynthesis is initiated by the gene *PDS1*, known to encode the enzyme p-hydroxyphenylpyruvate dioxygenase (HPPD) and was initially detected in carrot cells [10]. The corresponding *A. thaliana pds1* mutant was devoid of tocopherols and plastoquinones and had a lethal photobleached phenotype, thus showing that *PDS1* is a key gene of the tocopherol biosynthesis pathway [16,17]. In *Escherichia coli* (*E. coli)* expression studies, the protein HPPD catalyzed the accumulation of HGA. The subsequent oxidative polymerization of HGA to an ochronotic pigment resulted in a brownish coloration of the growth medium [10,80,81,82].

Constitutive expression of *PDS1* in *A. thaliana* plants led to elevated tocopherol concentrations of up to 43% in leaves and 28% in seeds, without a change in tocopherol composition [18]. In other studies, overexpression of the gene in tobacco leaves or in *A. thaliana* seeds resulted only in slightly increased tocopherol concentrations [83,84,85]. It was reasoned that HGA not only serves as a substrate for tocochromanols synthesis but also for other secondary plant metabolites e.g., plastoquinones [17]. Higher tocopherol concentrations were achieved when *PDS1* was co-expressed with downstream genes within the tocopherol biosynthetic pathway, the tocopherol content in seeds of *A. thaliana*, rapeseed, and corn kernels increased up to 1.8-, 2.0- and 3-fold, respectively [86,87,88].

The expression of *PDS1* is also linked to fruit ripening as well as leaf senescence in rice and barley [54,81,89]. Both ripening and leaf aging are correlated with an increase in tocopherol concentration, which is thought to be induced by ethylene treatment and oxidative stress [54,81]. This was underlined by sequence motifs for abscisic acid- and ethylene-specific response elements that were found in the promoter region of *PDS1*, indicating that these compounds can affect tocopherol biosynthesis.

Step 2: Polyprenyl Side Chain Synthesis

Uptake experiments of cotyledon-derived soybean suspension cultures with different tocopherol precursors demonstrated that PDP, together with HGA, has a high impact on tocopherol synthesis [86]. PDP, the polyprenyl side chain of tocopherols, can originate from two sources: direct reduction of geranylgeranyl diphosphate (GGDP) and free phytol, a product of chlorophyll degradation [14,90,91,92]. The latter was detected in experiments where the addition of free phytol in safflower cell cultures resulted in an 18-fold increase in total tocopherol content [93]. Additionally, feeding experiments of *A. thaliana* seedlings with labeled PDP demonstrated the integration of free phytol into tocopherols [92]. Further evidence was the detection of an *A. thaliana vte5*-1 mutant plant, which had a 20% reduced seed tocopherol concentration compared to the wild type and increased amounts of free phytol. The corresponding VTE5 protein had a high homology to a dolichol kinase and was able to catalyze the phosphorylation of free phytol into PDP [14].

Interestingly, tomato plants that have *VTE5*-like knockdown genes and are deficient in leaf and fruit tocopherol displayed multifaceted impacted metabolisms. While the chlorophyll content was not affected, the knockdown resulted in an alteration of the prenyl lipid profile in fruits and in an increase in the fatty acid phytyl ester synthesis in leaves, leading to changes in photosynthesis and sugar partitioning, which in turn affected tomato fruit quality [94].

The long-missing second enzyme of the phosphorylation pathway, phytyl phosphate kinase (VTE6) has recently been discovered using a phylogenetic approach [13]. The transgenic constitutive overexpression of the gene resulted in a 2-fold increase in PDP that led to a higher γ-tocopherol accumulation in seeds. The corresponding Arabidopsis mutant plants were tocopherol-deficient in leaves, and impaired in seed longevity and plant growth [13]. Intriguingly, the authors speculated that the impaired growth of the *vte6* mutants might be an effect of phytol accumulation rather than tocopherol shortage, resulting in a disruption of chlorophyll and galactolipid synthesis in chloroplast membranes. Recent studies also showed that tocopherol levels are indirectly regulated through the contribution of chlorophyll synthases by reducing PDP precursors, thus providing new possible alternative routes for improving tocopherol content [91,95].

Step 3: MPBQ (2-Methyl-6-Phytyl-1,4-Benzoquinol) Synthesis

The condensation of HGA and PDP to MPBQ is catalyzed by the enzyme HPT, which is encoded by the gene *VTE2* [96,97]. *A. thaliana* plants lacking *VTE2* are completely deficient in all tocopherol derivatives and all pathway precursors, indicating that this is a limiting step in tocopherol biosynthesis [98]. Transgenic approaches downregulating *VTE2* mRNA levels by antisense RNA led to a 10-fold reduction of tocopherol content in *A. thaliana* seeds. In contrast, overexpression of *VTE2* resulted in up to a 2-fold increased tocopherol concentration in seeds and up to a 4.4-fold increase in leaves [20,22]. Interestingly, in rapeseed, only minor increases were attained by seed-specific expression of the *VTE2* gene from the bacteria Synechocystis sp. PCC 6803, suggesting species-specific differences in tocopherol biosynthesis regulation [86]. The constitutive expression of the lettuce homolog (*LsHPT*) caused an increase up to 4-fold of the α- and 2.6-fold of γ-tocopherol content in lettuce leaves in comparison to the non-transformed plants [99].

The lack of tocopherols in *A. thaliana vte2* mutants made their functional role particularly apparent in those plants. Seedlings exhibited severe defects during germination and early seedling growth due to high concentrations of non-enzymatic lipid peroxides and hydroxy fatty acids, indicating that tocopherols are crucial for the prevention of lipid oxidation during these important developmental stages. The seedlings were disturbed in root growth and in cotyledon expansion and showed a slowed storage lipid catabolism [21]. Furthermore, the authors showed that seed viability was heavily impacted in *vte2* mutants compared to the wild type, probably due to the non-enzymatic oxidation of storage lipids. The accumulation of lipid peroxidation products in *vte2* mutants was correlated with an increased expression of many defense-related genes, showing another main role of tocopherols in the regulation of plant defense responses by modulating the levels of lipid peroxidation products [21]. The developmental and molecular phenotypes of *vte2* are abolished if tri-unsaturated fatty acids are suppressed as shown in the quadruple mutant *fad3-2fad7-2fad8vte2-1*, proving that there is a *vte2*-specific phenotype [100]. The presence of plastochromanol-8 (PC-8), a homolog of γ-tocotrienol with a C40 prenyl side chain, suppresses the phenotypes associated with lower plant fitness and seed longevity in *vte2* plants [101]. PC-8 has been found in vegetable oils and shown to display antioxidant activity, comparable to that of tocopherols [102,103].

Under normal growth conditions, as well as high light stress, adult *vte2* plants are indistinguishable from wild-type plants, confirming that tocopherols are substitutable by other antioxidants at this developmental stage [21,27,32]. However, if the same plants are subjected to low temperature, severe changes in gene transcription, as well as biochemical and physiological phenotypes, can be observed. The plants are compromised in transfer cell development and photoassimilate export, resulting in growth impairment [32,78,104]. Maeda et al., found that the specific underlying mechanisms need to be further investigated but speculate that plastid-localized tocopherols may have a role in the endoplasmic reticulum (ER) membrane lipid biosynthesis causing these effects [104].

Step 4: DMPBQ (2,3-dimethyl-6-phytyl-1,4-benzoquinone) Synthesis

The enzyme that methylates MPBQ to DMPBQ was first identified in *Synechocystis* sp. PCC 6803 and shares substrate specificities with the corresponding protein in *A. thaliana*, MPBQ methyltransferase [25,26]. The enzyme is encoded by the gene *VTE3* and without the catalysis of the prenylquinol DMPBQ, α- and γ-tocopherol could not be produced. The enzyme is also involved in plastoquinone synthesis and is able to use 2-methyl-6-solanesyl-1,4-benzoquinol (MSBQ) for the formation of plastoquinol-9 (PQ-9).

Several *A. thaliana vte3* mutants were isolated that had different phenotypes according to their origin and the severity of the mutation. Plants with a mutated *vet3-1* allele, derived from ethyl methanesulfonate (EMS) mutagenesis, were found to have a partial loss-of-function of the enzyme. As a result, an altered tocopherol composition along with reduced MPBQ/MSBQ methyltransferase activity was observed. In these plants, both δ- and β-tocopherol concentrations in leaves were increased at the expense of α-tocopherol, whereas γ-tocopherol was replaced by higher δ-tocopherol levels, but PQ amounts were not affected in seeds [25,26]. In contrast, plants with the non-functional *vte3-2* allele or Ds-tagged *A. thaliana* plants, which have a complete gene disruption, were deficient in α- and γ-tocopherol, as well as PQ. These mutants exhibited a pale green phenotype, abnormal chloroplasts and did not survive beyond the seedling stage [24,25].

The overexpression of *VTE3* in *A. thaliana* and soybean did not have a substantial impact on the tocopherol amounts but severely changed the tocopherol composition [26,75]. In seeds of transgenic soybean, increased γ- and α-tocopherol concentrations with simultaneous strong reductions in δ- and β-tocopherols concentrations were observed [26]. The seed-specific co-expression of *A. thaliana VTE3* and *VTE4* (γ-TMT) in soybean seeds shifted the tocopherol composition in favor of α-tocopherol (>90%). This is especially of interest for the genetic engineering of crop plants, in which seeds are naturally enriched in γ-tocopherol (e.g., rapeseed). The nutritional value of those plants could be enhanced by changing the composition towards α-tocopherol.

Step 5: Synthesis of γ-Tocopherol and δ-Tocopherol

Similar to the *vte2* mutant plants, *vte1* mutant plants lack all tocopherol forms due to the absence of a functional tocopherol cyclase (TC) enzyme. They accumulate the intermediate product DMPBQ, the un-cyclized precursor of γ-tocopherol, instead [27]. The expression of *AtVTE1* cDNA in *E. coli* efficiently converted DMPBQ into γ-tocopherol, demonstrating the cyclase activity of the enzyme [4,55,105]. Seed-specific expression of the *VTE1* gene from *A. thaliana* and maize (*Zea mays*) in transgenic rapeseed (*Brassica napus*) increased concentrations of all tocopherol forms in the seed oil of T_1_ and T_2_ generation plants. However, using different *B. napus VTE1* alleles, only a 1.5-fold increase in seed α-tocopherol was produced [82,105].

Interestingly, expression of the endogenous *VTE1* gene in *A. thaliana* plants under a constitutive promoter resulted in a 7-fold increase of the leaf tocopherol concentration and an extreme shift from α- to γ-tocopherol [28]. The reason for this shift was recently unraveled by Zbierzak et al. (2010). The authors showed that the co-elution of PC-8 with γ-tocopherol during High-performance liquid chromatography (HPLC) separation masked the result of increased γ-tocopherol concentrations [106]. PC-8 shares the same head group with γ-tocopherol and is derived from a cyclization step of PQ-9, the redox component of photosystem II. PQ-9 accumulates in *A. thaliana vte1* mutants, whereas PC-8 accumulates with tocopherols in *VTE1*-overexpressing plants, showing that TC is also able to cycle PQ-9 into PC-8 [105,106]. Accordingly, *AtVTE1* overexpression in rapeseed induces a 2.4-fold increase in PC-8 levels [105]. Nevertheless, the proportion of PC-8 with respect to total tocochromanols only ranged between 5 and 10%, suggesting that the majority of the effects observed in *vte1* mutants are indeed caused due to the absence of tocopherols [106].

The *A. thaliana vte1* mutant is not inhibited in its development, despite being deficient in tocopherols and ^1^O_2_ accumulation in chloroplasts [107]. Germination, growth, chlorophyll content, and photosynthetic quantum yield were similar to the wild type and only slightly different in response to photo-oxidative stress and/or low temperatures [21,27,108]. The absence of these phenotypes is explained by the accumulation of the redox-active DMPBQ, whose quinol form can have similar antioxidant properties to tocopherol [21]. Furthermore, the absence of tocopherols in *vte1* mutants resulted in an increase of ascorbate and glutathione, which belong to the most abundant antioxidants in higher plants. This effect was reversed when *VTE1* was overexpressed: their concentrations were reduced to 40–60% relative to wild-type plants [28,75]. It is known that the ascorbate–glutathione cycle is linked to the regeneration of tocopheroxyl radicals, the oxidized forms of tocopherol and the result of the reaction of tocopherols with lipid peroxyl radicals [36]. Hence, ascorbate deficiency in *A. thaliana vte1* mutants caused increased lipid peroxidation in water-stressed plants [109]. Furthermore, the overexpression of *VTE1* led to enhanced tolerance against salt stress in rice and drought stress in tobacco plants, providing evidence that crop species with increased tolerance to environmental stresses as well as high tocopherol content could be developed [110,111].

Intriguingly, another analysis of *A. thaliana vte1* mutants revealed a different role of tocopherols and indicated that α-tocopherol may have an impact on cellular signaling by altering plant hormone levels [112,113]. In these mutants, an age-dependent increase of jasmonic acid levels was detected. The levels were up to 2.4-fold higher compared to the wild type and accompanied by reduced plant growth and increased anthocyanin production. It is suggested that the reaction is indirectly triggered by tocopherols by controlling the extent of lipid peroxidation and therefore the accumulation of lipid hydroperoxides, which, in turn, are used for jasmonic acid synthesis [114]. The role of jasmonic acid, especially during senescence, is associated with the downregulation of housekeeping genes, such as photosynthetic genes, and with the upregulation of defense genes related to biotic and abiotic stress [115,116]. These findings further support the hypothesis that tocopherols may, albeit indirectly, contribute to gene expression regulation [78].

The analysis of the maize gene *sucrose export defective1* (*SXD1*) led to the suggestion that tocopherols might play a role in regulating carbon translocation. The maize *sxd1* mutant has a *vte1* mutant resembling phenotype that is characterized, amongst others, by the leaf-specific accumulation of starch and anthocyanin, and the deposition of callose in phloem parenchyma transfer cells. These characteristics are coincident with a loss of the symplastic transport, as well as a typical increase of the soluble sugar content due to the sucrose export deficiency [117,118,119]. RNAi inhibition of *StSXD1* expression in potato also induces defects in carbohydrate transport [120], showing that the functions of tocopherols are not assigned to a specific photosynthesis type and may be conserved in monocot and dicot plant species [55].

Step 6: Synthesis of α-Tocopherol and β-Tocopherol

The final step of the tocopherol synthesis is catalyzed by the gene *VTE4* and encodes a key enzyme (γ-TMT) that methylates both δ- and γ-tocopherol to β- and α-tocopherol, respectively (Figure 1). Correspondingly, α-tocopherol was absent in leaves of *vte4 A. thaliana* mutants, whereas high levels of γ-tocopherol accumulated [31]. Development and growth of the mutant plants was similar to that of the wild type, with only slight differences during oxidative stress, e.g., in chlorophyll content and photosynthetic quantum yield. The accumulation of γ-tocopherol had no impact on the amount of fatty acids or on lipid hydrolysis, indicating that γ-tocopherol is able to functionally substitute α-tocopherol [31,32].

*VTE4*-silenced tobacco plants had a decreased tolerance against salt-induced stress but an increased tolerance towards sorbitol stress and methyl viologen, an inductor of photo-oxidative stress. These findings suggest specific functions for different tocopherol forms with respect to stress, e.g., γ-tocopherol in the desiccation tolerance of seeds [50]. This hypothesis is supported by the fact that γ-tocopherol is the naturally predominant form in most seeds. Sattler et al. (2004) could show that a loss of γ-tocopherol increases the oxidation of polyunsaturated fatty acids and thereby diminishes seed longevity in *A. thaliana* [21]. Intriguingly, only in mature leaves of salt-stressed *vte4* mutant plants transcription levels of jasmonic acid- and ethylene-signaling genes were downregulated, but not in *vte1* mutants, or in wild-type plants. This suggests that γ-tocopherol, rather than α-tocopherol, plays a role in the regulation of these genes during osmotic stress [121].

The exclusive overexpression of the *VTE4* gene in leaves changed both tocopherol composition and content, resulting in elevated α-tocopherol values and up to a 30% increase in total tocopherol content [75]. Seed-specific expression of the gene nearly converted the entire amount of γ-tocopherol into α-tocopherol in soybean and *A. thaliana* [26,33]. Because most plant species accumulate mainly γ-tocopherol in seeds (>95%), γ-TMT activity might be a limiting bottleneck for α-tocopherol synthesis in seeds. This is consistent with the isolation of low α-tocopherol sunflower mutants, the natural wild type of which normally exhibit up to 90% α-tocopherol in kernels [122]. In these sunflower mutants, two *VTE4* paralogues are reduced or disrupted in their expression, leading to the accumulation of γ-tocopherol (>90%). This confirms that *VTE4* expression in sunflower seeds directly regulates the amount of α-tocopherol.

The fact that γ-TMT activity is limiting, becomes particularly clear when plants are exposed to abiotic stress. High levels of γ- and δ-tocopherol levels were observed in stressed leaves of *VTE2* overexpressing plants but not when *VTE2* and *VTE4* were simultaneously expressed [123]. Moreover, the concomitant overexpression of *VTE2* and *VTE4* was additive in *A. thaliana* leaves and seeds. The total tocopherol content increased up to 12-fold, while, at the same time, all of the γ-tocopherol was converted to α-tocopherol [20,75]. Thus, *VTE4* is suggested to be a key gene for metabolic engineering of enhanced α-tocopherol levels in crop plants. Similarly, using a transgenic approach, Endrigkeit et al., showed that a *B. napus VTE4* homolog was able to increase the α-tocopherol content 50-fold in transgenic Arabidopsis seeds, providing a promising candidate for the marker-assisted selection of α-tocopherol content in rapeseed [124].

## 5. Breeding for Higher Vitamin E Content

Improving vitamin E content in staple crops has been a major aim of crop breeding due to its benefits for health and oil quality [125]. Vitamin E content and composition vary widely in different crops, such as rapeseed, maize, rice, soybean, and barley [126,127,128,129,130]. These variations provide an incentive for breeding varieties that have superior vitamin E content. Quantitative genetic approaches, which map quantitative trait loci (QTLs) onto linkage maps or which detect associations between markers and phenotypes, are powerful methods to dissect complex traits in crops [131]. To date, dozens of QTL and association mapping studies related to vitamin E have been carried out in major crops including rapeseed, maize, soybean, rice, barley, and tomato [95,126,127,129,130,132,133,134]. As an example, Marwede et al., detected eight QTL-related to tocopherol content and composition in a doubled haploid rapeseed population [135]. However, Wang et al. discovered 33 QTLs and 61 associated loci for rapeseed tocopherol content and composition in a joint QTL, candidate gene, and association mapping study [132]. In another candidate-gene based association analysis, alleles of genes encoding the key enzymes of the core biosynthetic pathway associated with tocopherol content and composition in rapeseed were identified [126]. Intriguingly, the found QTLs explained only a small part of the phenotypic variation in tocopherol content and composition in rapeseed (5–30%). Diepenbrock et al. found 52 QTLs associated with vitamin E content using a 5000 line U.S. nested association mapping (NAM) panel [95]. Surprisingly, of the 14 resolved to individual genes, six novel genes affecting tocochromanols in plants were identified. These included unexpectedly two chlorophyll biosynthetic enzymes being major determinants of tocopherol content in non-photosynthetic maize grains, allowing new insights into tocopherol biosynthesis regulation [95]. Nevertheless, these QTL and association mapping analyses provide the foundation for improving vitamin E content in crops but also indicate that the genetic mechanism of vitamin E biosynthesis in crops remains incomplete and needs more comprehensive research. Some crop species are also facing breeding bottlenecks due to their low genetic inheritance in elite germplasm and have to use introgression from wild species, mutagenesis, or biotech methods to enhance different forms and levels of tocopherol [135,136,137,138].

Moreover, Quadrana et al., analyzed *VTE3* alleles and detected that their differential expression is associated with differences in methylation of a short-interspersed nuclear element (SINE) retrotransposon located in the promoter region that is responsible for the regulation of vitamin E content in tomatoes [139]. In another study, it was found that a chlorophyll synthase was able to induce the production of small interfering RNAs, accompanied with an increase in tocopherol levels [91]. These observations indicate that the tocopherol biosynthetic pathway might be regulated epigenetically and could also partially explain the low phenotypic variation found in QTL studies.

The vitamin E biosynthetic pathway has been well elucidated in model species, and select genes have been transformed and overexpressed individually or collectively in various plants to improve vitamin E content and composition [26,87,125,128,140,141,142,143,144,145,146,147,148,149]. For oil crops, the enhancement of γ-tocopherol is important in order to prevent oil peroxidation and thus to improve oil quality [150]. Since α-tocopherol is considered to have the highest nutritional value for humans and livestock, breeding crops with high α-tocopherol concentrations and good nutritional value is a target. For example, Yusuf et al., was able to elevate α-tocopherol concentrations 6-fold by overexpressing Arabidopsis *VTE4* in *B. juncea* plants [151]. In another study, α-tocopherol content was increased up to 11 times in soybean by overexpressing *VTE4* [152,153]. Interestingly, by transgenically expressing the *VTE1* gene, rice and tobacco plants with an increased tolerance against salt and drought stress, respectively, and a simultaneously enhanced tocopherol content were developed [110,111]. These findings provide prospects for breeding vitamin E biofortified crops that are even tolerant against environmental stresses through biotechnology methods.

In contrast to attempts to improve α-tocopherol content, it is relatively difficult to significantly increase the total vitamin E content in crops. The most successful attempts at increasing total vitamin E content have occurred in rapeseed and soybean, with a nearly 2.4-fold and 15-fold enhancement, respectively, of total vitamin E content after co-overexpression of several key genes for vitamin E biosynthesis [86,87]. Collectively, these observations indicate that the tocopherol biosynthetic pathway and its regulation are complex. Nevertheless, natural variations and transgenic strategies can be used in vitamin E biofortification breeding.

## 6. Conclusions and Challenges

Present studies using model plant species demonstrate that vitamin E biosynthesis genes are well understood. The use of mutant plants has helped with the discovery and analysis of the roles of tocopherols in plant processes beyond their antioxidant function. However, open questions remain about the specific functions of the different tocopherol forms as well as their concrete roles in signaling and defense responses. Additionally, plastoquinones and tocotrienols, which are known to be derived from the same head group as tocopherols, have gained attention in recent years but most of their roles still remain elusive [17,55,102,154,155].

Recently, new genes and epigenetic mechanisms have been discovered through investigations of the chlorophyll degradation pathway, transgenic and phylogenetic approaches, or by conducting joint linkage/genome-wide association studies [13,91,95]. Moreover, even in crops with hexaploid genomes, such as oat, the genes of the tocopherol pathway have been identified via deep sequencing and by using the high conservation of the tocopherol biosynthesis genes between plant species [156].

Natural variations of vitamin E content have been investigated and demonstrated wide variations in/among different crops, which suggest a tremendous genetic potential for breeding vitamin E improved crops. On this basis, several QTL/association mapping studies have been carried out and have detected a range of genetic loci associated with vitamin E content and composition, but of which many detected loci cannot pinpoint candidate genes [95,129,130,132,157,158,159]. Furthermore, epigenetics may be involved in regulating the vitamin E biosynthesis and can partially explain the low phenotypic variation of the detected genetic loci, indicating that vitamin E biosynthesis in crops may be more complex than that in model plants [91,139]. Transgenic approaches have also been used for vitamin E improvement in crops, and an increased α-tocopherol content has been achieved [20]. Elevating vitamin E content, even by stacking candidate genes, is still a challenge, but tocopherol enhancing alleles of the pathway genes have been identified, for example, in *B. napus*, and can now be used for marker-assisted breeding [86,126].

To date, many crops have been sequenced including vitamin E-rich staple crops, such as rapeseed, soybean, maize, and rice [160,161,162,163]. These sequences will be used in the process of cloning new genes from QTL and enable us to fully understand the genetic basis of vitamin E variations and biosynthesis. Thanks to the rapid development of biotechnology, many methods for efficient genome-editing have become available [164]. For example, CRISPR/Cas9 (Clustered Regularly Interspaced Short Palindromic Repeats/ CRISPR-associated protein 9), which is one of the most popular genome-editing methods at present, can efficiently and precisely edit gene/genes in mammals and plants. A genome-editing approach based on the cloned genes from QTL in crops and CRISPR/Cas9 can be a promising alternative strategy in breeding vitamin E biofortification crops [165].

## Figures and Tables

**Table 1:** Genes and enzymes of the tocopherol biosynthesis in *A. thaliana*.

Biosynthesis Step	Gene	Enzyme	Function	Substrate ^1^	Product	At Locus	Reference
1	*PDS1*	HPPD	Head group synthesis	HPP	HGA	AT1G06570	[16,17,18]
2	*VTE5*	Phytol kinase	Phosphorylation	Phytol + CTP/UTP	Phytyl-P	AT5G04490	[14]
	*VTE6*	Phytyl -P kinase	Phosphorylation	Phytol-P + CTP	Phytyl-PP(PDP)	AT1G78620	[13]
3	*VTE2*	HPT	Phytylation	HGA + PDP	MPBQ	AT2G18950	[19,20,21,22,23]
4	*VTE3*	MPBQ/MSBQ MT	Methylation	MPBQ/MSBQ	DMPQ	AT3G63410	[24,25,26]
5	*VTE1*	TC	Cyclization	MPBQ/DMPQ	γ-tocopherol/δ-tocopherol	AT4G32770	[27,28,29,30]
6	*VTE4*	γ-TMT	Methylation	δ-tocopherol/γ-tocopherol	α-tocopherol/β-tocopherol	AT1G64970	[30,31,32,33]

^1^ Only those compounds related to the tocopherol biosynthesis are mentioned. Genes and their corresponding enzyme names: *PDS1*/HPPD: p-hydroxyphenylpyruvate dioxygenase; *VTE1*/TC: tocopherol cyclase; *VTE2*/HPT: homogentisic acid phytyl transferase; *VTE3*/MT: methyltransferase; *VTE4*/ γ-TMT: γ-tocopherol methyltransferase; *VTE5/VTE6*: phytol kinase. Substrates and products: CTP/UTP: cytidine triphosphate/ uridine triphosphate; DMPBQ: 2,3-dimethyl-6-phytyl-1,4-benzoquinone; HGA: homogentisic acid; HPP: *p*-hydroxyphenylpyruvate; MPBQ: 2-methyl-6-phytyl-1,4-benzoquinol; MSBQ: 2-methyl-6-solanesyl-1,4-benzoquinol; Phytyl-PP/PDP: phytyl diphosphate; PDP: phytyldiphosphate.

## References

[1] DellaPenna D., Pogson B.J. (2006). Vitamin synthesis in plants: Tocopherols and carotenoids. Annu. Rev. Plant Biol..

[2] Grusak M.A., DellaPenna D. (1999). Improving the nutrient composition of plants to enhance human nutrition and health. Annu. Rev. Plant Biol..

[3] Munné-Bosch S., Alegre L. (2002). The function of tocopherols and tocotrienols in plants. Crit. Rev. Plant Sci..

[4] DellaPenna D., Last R.L. (2006). Progress in the dissection and manipulation of plant vitamin E biosynthesis. Physiol. Plant..

[5] DellaPenna D. (2005). A decade of progress in understanding vitamin E synthesis in plants. J. Plant Physiol..

[6] Péter S., Friedel A., Roos F.F., Wyss A., Eggersdorfer M., Hoffmann K., Weber P. (2016). A systematic review of global alpha-tocopherol status as assessed by nutritional intake levels and blood serum concentrations. Int. J. Vitam. Nutr. Res..

[7] Soll J., Douce R., Schultz G. (1980). Site of biosynthesis of alpha-tocopherol in spinach chloroplasts. FEBS Lett..

[8] Vidi P.A., Kanwischer M., Baginsky S., Austin J.R., Csucs G., Dormann P., Kessler F., Brehelin C. (2006). Tocopherol cyclase (VTE1) localization and vitamin E accumulation in chloroplast plastoglobule lipoprotein particles. J. Biol. Chem..

[9] Soll J., Schultz G., Joyard J., Douce R., Block M.A. (1985). Localization and synthesis of prenylquinones in isolated outer and inner envelope membranes from spinach chloroplasts. Arch. Biochem. Biophys..

[10] Garcia I., Rodgers M., Lenne C., Rolland A., Sailland A., Matringe M. (1997). Subcellular localization and purification of a *p*-hydroxyphenylpyruvate dioxygenase from cultured carrot cells and characterization of the corresponding cDNA. Biochem. J..

[11] Riewe D., Koohi M., Lisec J., Pfeiffer M., Lippmann R., Schmeichel J., Willmitzer L., Altmann T. (2012). A tyrosine aminotransferase involved in tocopherol synthesis in *Arabidopsis*. Plant J..

[12] Hortensteiner S. (2006). Chlorophyll degradation during senescence. Annu. Rev. Plant Biol..

[13] Vom Dorp K., Hölzl G., Plohmann C., Eisenhut M., Abraham M., Weber A.P., Hanson A.D., Dörmann P. (2015). Remobilization of phytol from chlorophyll degradation is essential for tocopherol synthesis and growth of *Arabidopsis*. Plant Cell.

[14] Valentin H.E., Lincoln K., Moshiri F., Jensen P.K., Qi Q., Venkatesh T.V., Karunanandaa B., Baszis S.R., Norris S.R., Savidge B. (2006). The Arabidopsis vitamin E pathway gene5-1 mutant reveals a critical role for phytol kinase in seed tocopherol biosynthesis. Plant Cell.

[15] Mene-Saffrane L., DellaPenna D. (2010). Biosynthesis, regulation and functions of tocochromanols in plants. Plant Physiol. Biochem..

[16] Norris S.R., Shen X., DellaPenna D. (1998). Complementation of the Arabidopsis pds1 mutation with the gene encoding *p*-hydroxyphenylpyruvate dioxygenase. Plant Physiol..

[17] Norris S.R., Barrette T.R., DellaPenna D. (1995). Genetic dissection of carotenoid synthesis in Arabidopsis defines plastoquinone as an essential component of phytoene desaturation. Plant Cell.

[18] Tsegaye Y., Shintani D.K., DellaPenna D. (2002). Overexpression of the enzyme *p*-hydroxyphenolpyruvate dioxygenase in *Arabidopsis* and its relation to tocopherol biosynthesis. Plant Physiol. Biochem..

[19] Schwab R., Palatnik J.F., Riester M., Schommer C., Schmid M., Weigel D. (2005). Specific effects of microRNAs on the plant transcriptome. Dev. Cell.

[20] Collakova E., DellaPenna D. (2003). Homogentisate phytyltransferase activity is limiting for tocopherol biosynthesis in *Arabidopsis*. Plant Physiol..

[21] Sattler S.E., Gilliland L.U., Magallanes-Lundback M., Pollard M., DellaPenna D. (2004). Vitamin E is essential for seed longevity and for preventing lipid peroxidation during germination. Plant Cell.

[22] Savidge B., Weiss J.D., Wong Y.H.H., Lassner M.W., Mitsky T.A., Shewmaker C.K., Post-Beittenmiller D., Valentin H.E. (2002). Isolation and characterization of homogentisate phytyltransferase genes from *Synechocystis* sp. PCC 6803 and *Arabidopsis*. Plant Physiol..

[23] Venkatesh T.V., Karunanandaa B., Free D.L., Rottnek J.M., Baszis S.R., Valentin H.E. (2006). Identification and characterization of an *Arabidopsis* homogentisate phytyltransferase paralog. Planta.

[24] Motohashi R., Ito T., Kobayashi M., Taji T., Nagata N., Asami T., Yoshida S., Yamaguchi-Shinozaki K., Shinozaki K. (2003). Functional analysis of the 37 kDa inner envelope membrane polypeptide in chloroplast biogenesis using a Ds-tagged *Arabidopsis* pale-green mutant. Plant J..

[25] Cheng Z., Sattler S., Maeda H., Sakuragi Y., Bryant D.A., DellaPenna D. (2003). Highly divergent methyltransferases catalyze a conserved reaction in tocopherol and plastoquinone synthesis in cyanobacteria and photosynthetic eukaryotes. Plant Cell.

[26] Van Eenennaam A.L., Lincoln K., Durrett T.P., Valentin H.E., Shewmaker C.K., Thorne G.M., Jiang J., Baszis S.R., Levering C.K., Aasen E.D. (2003). Engineering vitamin E content: From *Arabidopsis* mutant to soy oil. Plant Cell.

[27] Porfirova S., Bergmüller E., Tropf S., Lemke R., Dörmann P. (2002). Isolation of an *Arabidopsis* mutant lacking vitamin E and identification of a cyclase essential for all tocopherol biosynthesis. Proc. Natl. Acad. Sci. USA.

[28] Kanwischer M., Porfirova S., Bergmuller E., Dormann P. (2005). Alterations in tocopherol cyclase activity in transgenic and mutant plants of *Arabidopsis* affect tocopherol content, tocopherol composition, and oxidative stress. Plant Physiol..

[29] Sattler S.E., Cahoon E.B., Coughlan S.J., DellaPenna D. (2003). Characterization of tocopherol cyclases from higher plants and cyanobacteria. Evolutionary implications for tocopherol synthesis and function. Plant Physiol..

[30] Semchuk N.M., Lushchak O.V., Falk J., Krupinska K., Lushchak V.I. (2009). Inactivation of genes, encoding tocopherol biosynthetic pathway enzymes, results in oxidative stress in outdoor grown *Arabidopsis thaliana*. Plant Physiol. Biochem..

[31] Bergmüller E., Porfirova S., Dörmann P. (2003). Characterization of an *Arabidopsis* mutant deficient in γ-tocopherolmethyltransferase. Plant Mol. Biol..

[32] Maeda H., Song W., Sage T.L., DellaPenna D. (2006). Tocopherols play a crucial role in low-temperature adaptation and Phloem loading in *Arabidopsis*. Plant Cell.

[33] Shintani D., DellaPenna D. (1998). Elevating the vitamin E content of plants through metabolic engineering. Science.

[34] Brigelius-Flohe R., Traber M.G. (1999). Vitamin E: Function and metabolism. FASEB J..

[35] Krieger-Liszkay A., Fufezan C., Trebst A. (2008). Singlet oxygen production in photosystem II and related protection mechanism. Photosynth. Res..

[36] Kamal-Eldin A., Appelqvist L.A. (1996). The chemistry and antioxidant properties of tocopherols and tocotrienols. Lipids.

[37] Liebler D.C. (1993). The role of metabolism in the antioxidant function of vitamin E. Crit. Rev. Toxicol..

[38] Fukuzawa K., Tokumura A., Ouchi S., Tsukatani H. (1982). Antioxidant activities of tocopherols on Fe^2+^-ascorbate-induced lipid peroxidation in lecithin liposomes. Lipids.

[39] Halliwell B., Gutteridge J.M. (2015). Free Radicals in Biology and Medicine.

[40] Straight R.C., Spikes J.D., Frimer A.A. (1985). Photosensitized oxidation of biomolecules. Singlet O_2_.

[41] Lass A., Sohal R.S. (1998). Electron transport-linked ubiquinone-dependent recycling of alpha-tocopherol inhibits autooxidation of mitochondrial membranes. Arch. Biochem. Biophys..

[42] Siegel D., Bolton E.M., Burr J.A., Liebler D.C., Ross D. (1997). The reduction of α-tocopherolquinone by human NAD(P)H: Quinone oxidoreductase: The role of α-tocopherolhydroquinone as a cellular antioxidant. Mol. Pharmacol..

[43] Wu J.H., Croft K.D. (2007). Vitamin E metabolism. Mol. Aspects Med..

[44] Horvath G., Wessjohann L., Bigirimana J., Jansen M., Guisez Y., Caubergs R., Horemans N. (2006). Differential distribution of tocopherols and tocotrienols in photosynthetic and non-photosynthetic tissues. Phytochemistry.

[45] Velasco L., Fernández-Martínez J., Garcia-Ruiz R., Domínguez J. (2002). Genetic and environmental variation for tocopherol content and composition in sunflower commercial hybrids. J. Agric. Sci..

[46] Bruni R., Muzzoli M., Ballero M., Loi M.C., Fantin G., Poli F., Sacchetti G. (2004). Tocopherols, fatty acids and sterols in seeds of four Sardinian wild Euphorbia species. Fitoterapia.

[47] Horvath G., Wessjohann L., Bigirimana J., Monica H., Jansen M., Guisez Y., Caubergs R., Horemans N. (2006). Accumulation of tocopherols and tocotrienols during seed development of grape (*Vitis vinifera* L. cv. Albert Lavallee). Plant Physiol. Biochem..

[48] Cayuela J.A., García J.F. (2017). Sorting olive oil based on alpha-tocopherol and total tocopherol content using near-infra-red spectroscopy (NIRS) analysis. J. Food Eng..

[49] Gotor A.A., Farkas E., Berger M., Labalette F., Centis S., Daydé J., Calmon A. (2007). Determination of tocopherols and phytosterols in sunflower seeds by NIR spectrometry. Eur. J. Lipid Sci. Technol..

[50] Abbasi A.R., Hajirezaei M., Hofius D., Sonnewald U., Voll L.M. (2007). Specific roles of alpha- and gamma-tocopherol in abiotic stress responses of transgenic tobacco. Plant Physiol..

[51] Arango Y., Heise K.-P. (1998). Localization of α-tocopherol synthesis in chromoplast envelope membranes of *Capsicum annuum* L. fruits. J. Exp. Bot..

[52] Abushita A.A., Hebshi E.A., Daood H.G., Biacs P.A. (1997). Determination of antioxidant vitamins in tomatoes. Food Chem..

[53] Arrom L., Munné-Bosch S. (2010). Tocopherol composition in flower organs of *Lilium* and its variations during natural and artificial senescence. Plant Sci..

[54] Falk J., Krauß N., Dähnhardt D., Krupinska K. (2002). The senescence associated gene of barley encoding 4-hydroxyphenylpyruvate dioxygenase is expressed during oxidative stress. J. Plant Physiol..

[55] Falk J., Munne-Bosch S. (2010). Tocochromanol functions in plants: Antioxidation and beyond. J. Exp. Bot..

[56] Evans H.M., Bishop K.S. (1922). On the Existence of a Hitherto Unrecognized Dietary Factor Essential for Reproduction. Science.

[57] Azzi A., Gysin R., Kempna P., Munteanu A., Negis Y., Villacorta L., Visarius T., Zingg J.M. (2004). Vitamin E mediates cell signaling and regulation of gene expression. Ann. N. Y. Acad. Sci..

[58] Azzi A., Ricciarelli R., Zingg J.M. (2002). Non-antioxidant molecular functions of α-tocopherol (vitamin E). FEBS Lett..

[59] Zingg J.M., Azzi A. (2004). Non-antioxidant activities of vitamin E. Curr. Med. Chem..

[60] Schneider C. (2005). Chemistry and biology of vitamin E. Mol. Nutr. Food Res..

[61] Kushi L.H., Folsom A.R., Prineas R.J., Mink P.J., Wu Y., Bostick R.M. (1996). Dietary antioxidant vitamins and death from coronary heart disease in postmenopausal women. N. Engl. J. Med..

[62] Weinstein S.J., Wright M.E., Lawson K.A., Snyder K., Mannisto S., Taylor P.R., Virtamo J., Albanes D. (2007). Serum and dietary vitamin E in relation to prostate cancer risk. Cancer Epidemiol. Biomark. Prev..

[63] Bramley P.M., Elmadfa I., Kafatos A., Kelly F.J., Manios Y., Roxborough H.E., Schuch W., Sheehy P.J.A., Wagner K.H. (2000). Vitamin E. J. Sci. Food Agric..

[64] Öhrvall M., Vessby B., Sundlöf G. (1996). Gamma, but not alpha, tocopherol levels in serum are reduced in coronary heart disease patients. J. Intern. Med..

[65] Schuelke M., Mayatepek E., Inter M., Becker M., Pfeiffer E., Speer A., Hubner C., Finckh B. (1999). Treatment of ataxia in isolated vitamin E deficiency caused by alpha-tocopherol transfer protein deficiency. J. Pediatr..

[66] Witztum J.L., Steinberg D. (1991). Role of oxidized low density lipoprotein in atherogenesis. J. Clin. Investig..

[67] Traber M.G., Frei B., Beckman J.S. (2008). Vitamin E revisited: Do new data validate benefits for chronic disease prevention?. Curr. Opin. Lipidol..

[68] Niki E. (2010). Do free radicals play causal role in atherosclerosis? Low density lipoprotein oxidation and vitamin E revisited. J. Clin. Biochem. Nutr..

[69] Colombo M.L. (2010). An update on vitamin E, tocopherol and tocotrienol-perspectives. Molecules.

[70] Azzi A. (2017). Many tocopherols, one vitamin E. Mol. Asp. Med..

[71] Azzi A., Meydani S.N., Meydani M., Zingg J.M. (2016). The rise, the fall and the renaissance of vitamin E. Arch. Biochem. Biophys..

[72] Jiang Q. (2014). Natural forms of vitamin E: Metabolism, antioxidant, and anti-inflammatory activities and their role in disease prevention and therapy. Free Radic. Biol. Med..

[73] Dietrich M., Traber M.G., Jacques P.F., Cross C.E., Hu Y., Block G. (2006). Does γ-tocopherol play a role in the primary prevention of heart disease and cancer? A review. J. Am. Coll. Nutr..

[74] Kloz M., Pillai S., Kodis G., Gust D., Moore T.A., Moore A.L., van Grondelle R., Kennis J.T. (2011). Carotenoid photoprotection in artificial photosynthetic antennas. J. Am. Chem. Soc..

[75] Li Y., Zhou Y., Wang Z.A., Sun X.F., Tang K.X. (2010). Engineering tocopherol biosynthetic pathway in *Arabidopsis* leaves and its effect on antioxidant metabolism. Plant Sci..

[76] Asensi-Fabado M.A., Munne-Bosch S. (2010). Vitamins in plants: Occurrence, biosynthesis and antioxidant function. Trends Plant Sci..

[77] Azzi A. (2007). Molecular mechanism of α-tocopherol action. Free Radic. Biol. Med..

[78] Sattler S.E., Mene-Saffrane L., Farmer E.E., Krischke M., Mueller M.J., DellaPenna D. (2006). Nonenzymatic lipid peroxidation reprograms gene expression and activates defense markers in *Arabidopsis* tocopherol-deficient mutants. Plant Cell.

[79] Hyun T.K., Kumar K., Rao K.P., Sinha A.K., Roitsch T. (2011). Role of α-tocopherol in cellular signaling: α-tocopherol inhibits stress-induced mitogen-activated protein kinase activation. Plant Biotechnol. Rep..

[80] Denoya C.D., Skinner D.D., Morgenstern M.R. (1994). A *Streptomyces avermitilis* gene encoding a 4-hydroxyphenylpyruvic acid dioxygenase-like protein that directs the production of homogentisic acid and an ochronotic pigment in *Escherichia coli*. J. Bacteriol..

[81] Singh R.K., Ali S.A., Nath P., Sane V.A. (2011). Activation of ethylene-responsive *p*-hydroxyphenylpyruvate dioxygenase leads to increased tocopherol levels during ripening in mango. J. Exp. Bot..

[82] Fritsche S., Wang X., Nichelmann L., Suppanz I., Hadenfeldt S., Endrigkeit J., Meng J., Jung C. (2014). Genetic and functional analysis of tocopherol biosynthesis pathway genes from rapeseed (*Brassica napus* L.). Plant Breed..

[83] Falk J., Andersen G., Kernebeck B., Krupinska K. (2003). Constitutive overexpression of barley 4-hydroxyphenylpyruvate dioxygenase in tobacco results in elevation of the vitamin E content in seeds but not in leaves. FEBS Lett..

[84] Rippert P., Scimemi C., Dubald M., Matringe M. (2004). Engineering plant shikimate pathway for production of tocotrienol and improving herbicide resistance. Plant Physiol..

[85] Li Y., Wang Z., Sun X., Tang K. (2008). Current opinions on the functions of tocopherol based on the genetic manipulation of tocopherol biosynthesis in plants. J. Integr. Plant Biol..

[86] Karunanandaa B., Qi Q., Hao M., Baszis S.R., Jensen P.K., Wong Y.H., Jiang J., Venkatramesh M., Gruys K.J., Moshiri F. (2005). Metabolically engineered oilseed crops with enhanced seed tocopherol. Metab. Eng..

[87] Raclaru M., Gruber J., Kumar R., Sadre R., Lühs W., Zarhloul M., Friedt W., Frentzen M., Weier D. (2006). Increase of the tocochromanol content in transgenic *Brassica napus* seeds by overexpression of key enzymes involved in prenylquinone biosynthesis. Mol. Breed..

[88] Naqvi S., Farre G., Zhu C., Sandmann G., Capell T., Christou P. (2011). Simultaneous expression of *Arabidopsis* rho-hydroxyphenylpyruvate dioxygenase and MPBQ methyltransferase in transgenic corn kernels triples the tocopherol content. Transgenic Res..

[89] Kleber-Janke T., Krupinska K. (1997). Isolation of cDNA clones for genes showing enhanced expression in barley leaves during dark-induced senescence as well as during senescence under field conditions. Planta.

[90] Keller Y., Bouvier F., D’Harlingue A., Camara B. (1998). Metabolic compartmentation of plastid prenyllipid biosynthesis—Evidence for the involvement of a multifunctional geranylgeranyl reductase. Eur. J. Biochem..

[91] Zhang C., Zhang W., Ren G., Li D., Cahoon R.E., Chen M., Zhou Y., Yu B., Cahoon E.B. (2015). Chlorophyll synthase under epigenetic surveillance is critical for vitamin E synthesis, and altered expression affects tocopherol levels in *Arabidopsis*. Plant Physiol..

[92] Ischebeck T., Zbierzak A.M., Kanwischer M., Dormann P. (2006). A salvage pathway for phytol metabolism in *Arabidopsis*. J. Biol. Chem..

[93] Furuya T., Yoshikawa T., Kimura T., Kaneko H. (1987). Production of tocopherols by cell culture of safflower. Phytochemistry.

[94] Almeida J., Azevedo Mda S., Spicher L., Glauser G., vom Dorp K., Guyer L., del Valle Carranza A., Asis R., de Souza A.P., Buckeridge M. (2016). Down-regulation of tomato PHYTOL KINASE strongly impairs tocopherol biosynthesis and affects prenyllipid metabolism in an organ-specific manner. J. Exp. Bot..

[95] Diepenbrock C.H., Kandianis C.B., Lipka A.E., Magallanes-Lundback M., Vaillancourt B., Gongora-Castillo E., Wallace J.G., Cepela J., Mesberg A., Bradbury P. (2017). Novel Loci Underlie Natural Variation in Vitamin E Levels in Maize Grain. Plant Cell.

[96] Collakova E., DellaPenna D. (2001). Isolation and functional analysis of homogentisate phytyltransferase from *Synechocystis* sp. PCC 6803 and *Arabidopsis*. Plant Physiol..

[97] Schledz M., Seidler A., Beyer P., Neuhaus G. (2001). A novel phytyltransferase from *Synechocystis* sp. PCC 6803 involved in tocopherol biosynthesis. FEBS Lett..

[98] Sattler S.E., Cheng Z., DellaPenna D. (2004). From *Arabidopsis* to agriculture: Engineering improved Vitamin E content in soybean. Trends Plant Sci..

[99] Ren W., Zhao L., Wang Y., Cui L., Tang Y., Sun X., Tang K. (2011). Overexpression of homogentisate phytyltransferase in lettuce results in increased content of vitamin E. AJB.

[100] Mene-Saffrane L., Davoine C., Stolz S., Majcherczyk P., Farmer E.E. (2007). Genetic removal of tri-unsaturated fatty acids suppresses developmental and molecular phenotypes of an *Arabidopsis* tocopherol-deficient mutant. Whole-body mapping of malondialdehyde pools in a complex eukaryote. J. Biol. Chem..

[101] Mene-Saffrane L., Jones A.D., DellaPenna D. (2010). Plastochromanol-8 and tocopherols are essential lipid-soluble antioxidants during seed desiccation and quiescence in *Arabidopsis*. Proc. Natl. Acad. Sci. USA.

[102] Ksas B., Becuwe N., Chevalier A., Havaux M. (2015). Plant tolerance to excess light energy and photooxidative damage relies on plastoquinone biosynthesis. Sci. Rep..

[103] Olejnik D., Gogolewski M., Nogala-Kałucka M. (1997). Isolation and some properties of plastochromanol-8. Mol. Nutr. Food Res..

[104] Maeda H., Song W., Sage T., Dellapenna D. (2014). Role of callose synthases in transfer cell wall development in tocopherol deficient *Arabidopsis* mutants. Front. Plant Sci..

[105] Kumar R., Raclaru M., Schusseler T., Gruber J., Sadre R., Lühs W., Zarhloul K.M., Friedt W., Enders D., Frentzen M. (2005). Characterisation of plant tocopherol cyclases and their overexpression in transgenic *Brassica napus* seeds. FEBS Lett..

[106] Zbierzak A.M., Kanwischer M., Wille C., Vidi P.A., Giavalisco P., Lohmann A., Briesen I., Porfirova S., Brehelin C., Kessler F. (2010). Intersection of the tocopherol and plastoquinol metabolic pathways at the plastoglobule. Biochem. J..

[107] Rastogi A., Yadav D.K., Szymanska R., Kruk J., Sedlarova M., Pospisil P. (2014). Singlet oxygen scavenging activity of tocopherol and plastochromanol in *Arabidopsis thaliana*: Relevance to photooxidative stress. Plant Cell Environ..

[108] Havaux M., Eymery F., Porfirova S., Rey P., Dormann P. (2005). Vitamin E protects against photoinhibition and photooxidative stress in *Arabidopsis thaliana*. Plant Cell.

[109] Munné-Bosch S., Alegre L. (2002). Interplay between ascorbic acid and lipophilic antioxidant defences in chloroplasts of water-stressed *Arabidopsis* plants. FEBS Lett..

[110] Liu X., Hua X., Guo J., Qi D., Wang L., Liu Z., Jin Z., Chen S., Liu G. (2008). Enhanced tolerance to drought stress in transgenic tobacco plants overexpressing VTE1 for increased tocopherol production from *Arabidopsis thaliana*. Biotechnol. Lett..

[111] Ouyang S., He S., Liu P., Zhang W., Zhang J., Chen S. (2011). The role of tocopherol cyclase in salt stress tolerance of rice (*Oryza sativa*). Sci. China Life Sci..

[112] Munne-Bosch S. (2005). Linking tocopherols with cellular signaling in plants. New Phytol..

[113] Munné-Bosch S., Weiler E.W., Alegre L., Müller M., Düchting P., Falk J. (2007). α-Tocopherol may influence cellular signaling by modulating jasmonic acid levels in plants. Planta.

[114] Schaller F. (2001). Enzymes of the biosynthesis of octadecanoid-derived signalling molecules. J. Exp. Bot..

[115] Creelman R.A., Mullet J.E. (1997). Biosynthesis and Action of Jasmonates in Plants. Annu. Rev. Plant Physiol. Plant Mol. Biol..

[116] Wasternack C. (2007). Jasmonates: An update on biosynthesis, signal transduction and action in plant stress response, growth and development. Ann. Bot..

[117] Botha C., Cross R., Van Bel A., Peter C. (2000). Phloem loading in the sucrose-export-defective (SXD-1) mutant maize is limited by callose deposition at plasmodesmata in bundle sheath—Vascular parenchyma interface. Protoplasma.

[118] Provencher L.M., Miao L., Sinha N., Lucas W.J. (2001). Sucrose export defective1 encodes a novel protein implicated in chloroplast-to-nucleus signaling. Plant Cell.

[119] Russin W.A., Evert R.F., Vanderveer P.J., Sharkey T.D., Briggs S.P. (1996). Modification of a Specific Class of Plasmodesmata and Loss of Sucrose Export Ability in the sucrose export defective1 Maize Mutant. Plant Cell.

[120] Hofius D., Hajirezaei M.R., Geiger M., Tschiersch H., Melzer M., Sonnewald U. (2004). RNAi-mediated tocopherol deficiency impairs photoassimilate export in transgenic potato plants. Plant Physiol..

[121] Cela J., Chang C., Munné-Bosch S. (2011). Accumulation of γ-rather than α-tocopherol alters ethylene signaling gene expression in the *vte4* mutant of *Arabidopsis thaliana*. Plant Cell Physiol..

[122] Hass C.G., Tang S., Leonard S., Traber M.G., Miller J.F., Knapp S.J. (2006). Three non-allelic epistatically interacting methyltransferase mutations produce novel tocopherol (vitamin E) profiles in sunflower. Theor. Appl. Genet..

[123] Collakova E., DellaPenna D. (2003). The role of homogentisate phytyltransferase and other tocopherol pathway enzymes in the regulation of tocopherol synthesis during abiotic stress. Plant Physiol..

[124] Endrigkeit J., Wang X., Cai D., Zhang C., Long Y., Meng J., Jung C. (2009). Genetic mapping, cloning, and functional characterization of the *BnaX.VTE4* gene encoding a gamma-tocopherol methyltransferase from oilseed rape. Theor. Appl. Genet..

[125] Hunter S.C., Cahoon E.B. (2007). Enhancing vitamin E in oilseeds: Unraveling tocopherol and tocotrienol biosynthesis. Lipids.

[126] Fritsche S., Wang X., Li J., Stich B., Kopisch-Obuch F.J., Endrigkeit J., Leckband G., Dreyer F., Friedt W., Meng J. (2012). A candidate gene-based association study of tocopherol content and composition in rapeseed (*Brassica napus*). Front Plant Sci..

[127] Muzhingi T., Palacios-Rojas N., Miranda A., Cabrera M.L., Yeum K.J., Tang G. (2017). Genetic variation of carotenoids, vitamin E and phenolic compounds in Provitamin A biofortified maize. J. Sci. Food Agric..

[128] Wang X.Q., Yoon M.Y., He Q., Kim T.S., Tong W., Choi B.W., Lee Y.S., Park Y.J. (2015). Natural variations in OsgammaTMT contribute to diversity of the alpha-tocopherol content in rice. Mol. Genet. Genomics.

[129] Shaw E.J., Rajcan I., Morris B. (2017). Molecular mapping of soybean seed tocopherols in the cross ‘OAC Bayfield’ × ‘OAC Shire’. Plant Breed..

[130] Graebner R.C., Wise M., Cuesta-Marcos A., Geniza M., Blake T., Blake V.C., Butler J., Chao S., Hole D.J., Horsley R. (2015). Quantitative Trait Loci Associated with the Tocochromanol (Vitamin E) Pathway in Barley. PLoS ONE.

[131] Mauricio R. (2001). Mapping quantitative trait loci in plants: Uses and caveats for evolutionary biology. Nat. Rev. Genet..

[132] Wang X., Zhang C., Li L., Fritsche S., Endrigkeit J., Zhang W., Long Y., Jung C., Meng J. (2012). Unraveling the genetic basis of seed tocopherol content and composition in rapeseed (*Brassica napus* L.). PLoS ONE.

[133] Lipka A.E., Gore M.A., Magallanes-Lundback M., Mesberg A., Lin H., Tiede T., Chen C., Buell C.R., Buckler E.S., Rocheford T. (2013). Genome-wide association study and pathway-level analysis of tocochromanol levels in maize grain. G3 (Bethesda).

[134] Luby C.H., Maeda H.A., Goldman I.L. (2014). Genetic and phenological variation of tocochromanol (vitamin E) content in wild (*Daucus carota* L. var. carota) and domesticated carrot (*D. carota* L. var. sativa). Hortic. Res..

[135] Rani R., Sheoran R., Sharma B. (2017). Perspectives of breeding for altering sunflower oil quality to obtain novel oils. Int. J. Curr. Microbiol. App. Sci..

[136] Rauf S., Jamil N., Tariq S.A., Khan M., Kausar M., Kaya Y. (2017). Progress in modification of sunflower oil to expand its industrial value. J. Sci. Food Agric..

[137] Seyis F., Snowdon R., Luhs W., Friedt W. (2003). Molecular characterization of novel resynthesized rapeseed (*Brassica napus)* lines and analysis of their genetic diversity in comparison with spring rapeseed cultivars. Plant Breed..

[138] Hasan M., Seyis F., Badani A., Pons-Kühnemann J., Friedt W., Lühs W., Snowdon R.J. (2006). Analysis of genetic diversity in the *Brassica napus* L. gene pool using SSR markers. Genet. Resour. Crop Evol..

[139] Quadrana L., Almeida J., Asis R., Duffy T., Dominguez P.G., Bermudez L., Conti G., Correa da Silva J.V., Peralta I.E., Colot V. (2014). Natural occurring epialleles determine vitamin E accumulation in tomato fruits. Nat. Commun..

[140] Farre G., Sudhakar D., Naqvi S., Sandmann G., Christou P., Capell T., Zhu C. (2012). Transgenic rice grains expressing a heterologous rho-hydroxyphenylpyruvate dioxygenase shift tocopherol synthesis from the gamma to the alpha isoform without increasing absolute tocopherol levels. Transgenic Res..

[141] Chaudhary N., Khurana P. (2013). Cloning, functional characterisation and transgenic manipulation of vitamin E biosynthesis genes of wheat. Funct. Plant Biol..

[142] Espinoza A., San Martin A., Lopez-Climent M., Ruiz-Lara S., Gomez-Cadenas A., Casaretto J.A. (2013). Engineered drought-induced biosynthesis of alpha-tocopherol alleviates stress-induced leaf damage in tobacco. J. Plant Physiol..

[143] Chen D., Chen H., Zhang L., Shi X., Chen X. (2014). Tocopherol-deficient rice plants display increased sensitivity to photooxidative stress. Planta.

[144] Jin S., Daniell H. (2014). Expression of gamma-tocopherol methyltransferase in chloroplasts results in massive proliferation of the inner envelope membrane and decreases susceptibility to salt and metal-induced oxidative stresses by reducing reactive oxygen species. Plant Biotechnol. J..

[145] Tanaka H., Yabuta Y., Tamoi M., Tanabe N., Shigeoka S. (2015). Generation of transgenic tobacco plants with enhanced tocotrienol levels through the ectopic expression of rice homogentisate geranylgeranyl transferase. Plant Biotechnol. J..

[146] Che P., Zhao Z.Y., Glassman K., Dolde D., Hu T.X., Jones T.J., Obukosia S., Wambugu F., Albertsen M.C. (2016). Elevated vitamin E content improves all-trans beta-carotene accumulation and stability in biofortified sorghum. Proc. Natl. Acad. Sci. USA.

[147] Levac D., Cázares P., Yu F., De Luca V. (2016). A picrinine N-methyltransferase belongs to a new family of γ-tocopherol-like methyltransferases found in medicinal plants that make biologically active monoterpenoid indole alkaloids. Plant Physiol..

[148] Fleta-Soriano E., Munne-Bosch S. (2017). Enhanced plastochromanol-8 accumulation during reiterated drought in maize (*Zea mays* L.). Plant Physiol. Biochem..

[149] Liao P., Chen X., Wang M., Bach T.J., Chye M.L. (2017). Improved fruit α-tocopherol, carotenoid, squalene and phytosterol contents through manipulation of Brassica juncea 3-HYDROXY-3-METHYLGLUTARYL-COA SYNTHASE1 in transgenic tomato. Plant Biotechnol. J..

[150] Demurin Y., Skoric D., Karlovic D. (1996). Genetic variability of tocopherol composition in sunflower seeds as a basis of breeding improved oil quality. Plant Breed..

[151] Yusuf M.A., Sarin N.B. (2007). Antioxidant value addition in human diets: Genetic transformation of Brassica juncea with gamma-TMT gene for increased alpha-tocopherol content. Transgenic Res..

[152] Vinutha T., Maheswari C., Bansal N., Prashat G.R., Krishnan V., Kumari S., Dahuja A., Sachdev A., Rai R. (2015). Expression analysis of γ-tocopherol methyl transferase genes and α-tocopherol content in developing seeds of soybean [*Glycine max* (L.) Merr.]. IJBB.

[153] Tavva V.S., Kim Y.H., Kagan I.A., Dinkins R.D., Kim K.H., Collins G.B. (2007). Increased alpha-tocopherol content in soybean seed overexpressing the *Perilla frutescens* gamma-tocopherol *methyltransferase* gene. Plant Cell. Rep..

[154] Siles L., Cela J., Munne-Bosch S. (2013). Vitamin E analyses in seeds reveal a dominant presence of tocotrienols over tocopherols in the Arecaceae family. Phytochemistry.

[155] Matringe M., Ksas B., Rey P., Havaux M. (2008). Tocotrienols, the unsaturated forms of vitamin E, can function as antioxidants and lipid protectors in tobacco leaves. Plant Physiol..

[156] Gutierrez-Gonzalez J.J., Garvin D.F. (2016). Subgenome-specific assembly of vitamin E biosynthesis genes and expression patterns during seed development provide insight into the evolution of oat genome. Plant Biotechnol. J..

[157] Marwede V., Gul M.K., Becker H.C., Ecke W. (2005). Mapping of QTL controlling tocopherol content in winter oilseed rape. Plant Breed..

[158] Li H.Y., Liu H.C., Han Y.P., Wu X.X., Teng W.L., Liu G.F., Li W.B. (2010). Identification of QTL underlying vitamin E contents in soybean seed among multiple environments. Theor. Appl. Genet..

[159] Liu H., Cao G., Wu D., Jiang Z., Han Y., Li W., Morris B. (2017). Quantitative trait loci underlying soybean seed tocopherol content with main additive, epistatic and QTL × environment effects. Plant Breed..

[160] Ding Y., Li H., Chen L.L., Xie K. (2016). Recent Advances in Genome Editing Using CRISPR/Cas9. Front. Plant Sci..

[161] Doudna J.A., Gersbach C.A. (2015). Genome editing: The end of the beginning. Genome Biol..

[162] Feng Z., Zhang B., Ding W., Liu X., Yang D.L., Wei P., Cao F., Zhu S., Zhang F., Mao Y. (2013). Efficient genome editing in plants using a CRISPR/Cas system. Cell Res..

[163] Gao W., Long L., Tian X., Xu F., Liu J., Singh P.K., Botella J.R., Song C. (2017). Genome Editing in Cotton with the CRISPR/Cas9 System. Front. Plant Sci..

[164] Cardi T., Neal Stewart C. (2016). Progress of targeted genome modification approaches in higher plants. Plant Cell Rep..

[165] Jung C., Capistrano-Gossmann G., Braatz J., Sashidhar N., Melzer S. (2017). Recent developments in genome editing and applications in plant breeding. Plant Breed..

